# Inequity in Assessment Among Pediatric Residents

**DOI:** 10.1001/jamanetworkopen.2025.5594

**Published:** 2025-04-17

**Authors:** Hannah L. Kakara Anderson, Daniel C. West, Alan J. Schwartz, Anna K. Weiss, Carolyn Marcus, Elizabeth Hanson, David A. Turner, Daniel J. Schumacher

**Affiliations:** 1 The Children’s Hospital of Philadelphia (CHOP), Philadelphia, Pennsylvania; 2 Perelman School of Medicine at the University of Pennsylvania, Philadelphia; 3 Maastricht University School of Health Professions Education, Maastricht, the Netherlands; 4 CHOP Education Collaboratory, Philadelphia, Pennsylvania; 5 Association of Pediatric Program Directors Longitudinal Educational Assessment Research Network, McLean, Virginia; 6 Boston Children’s Hospital, Boston, Massachusetts; 7 Joe R. and Teresa Lozano Long School of Medicine, University of Texas Health San Antonio; 8 American Board of Pediatrics, Chapel Hill, North Carolina; 9 Cincinnati Children’s Hospital Medical Center, Cincinnati, Ohio; 10 University of Cincinnati College of Medicine, Cincinnati, Ohio

## Abstract

This survey study evaluates self-reported experiences of biased verbal and written assessments in medical education.

## Introduction

In undergraduate and graduate medical education (GME), verbal and written assessments provide feedback to learners and inform decisions about advancement.^1^ These assessments are prone to inequity, reflecting biases leading to disparities in performance assessment.^2^ Disparities in scores and differential, biased language in written and verbal assessments of learners underrepresented in medicine (URIM) and female learners have been described in undergraduate medical education and several GME specialties, but not pediatrics.^2,3^ Understanding whether these inequities exist in pediatrics is important because inequities accumulate over time, harming trainees’ career trajectories and society’s interest in a diverse physician workforce.^4,5^ In this study, we sought to determine whether pediatric residents had experienced inequity in assessments.

## Methods

From May 2023 to June 2024 as part of a multicenter study, we surveyed residents in 15 US pediatric residency programs. Cincinnati Children’s Institutional Review Board deemed this survey study exempt from review due to minimal risk survey procedures. Participants provided written consent. We followed the AAPOR reporting guideline.

We asked residents for their demographic characteristics, whether they had ever experienced inequity in written or verbal assessment, and if so, how frequently (eAppendix in Supplement 1). Demographic groups included race and ethnicity (Asian, URIM [African American or Black, American Indian or Alaskan Native, Hispanic, Native Hawaiian or Other Pacific Islander], White), gender identity, sexual orientation, disability identity, medical degree (international or American medical graduate [IMG/AMG]), and socioeconomic status. We defined ever experiencing inequity in assessment as reporting inequity in either written or verbal assessments and frequently experiencing inequity in assessment as responding sometimes, usually, or almost always.

We compared reported frequency of inequities across demographic groups using the Pearson χ^2^ test. We then modeled the association between demographic groups (plus 2 intersectional groups: URIM-female, Asian-female) and 2 dependent variables (ever and frequently experiencing inequity in assessment) using multivariable logistic regression. To determine intersectional relationships, we combined interactions between the 2 dependent variables with main outcomes. We included a random program intercept to adjust for residents clustered in programs. Data analysis was performed with R 4.0.2 (R Core Team).

## Results

Overall, 479 of 803 pediatric residents (59.7%) completed the survey (345 females (72.0%); 108 Asian [22.6%], 82 URIM [17.1%], 289 White [60.3%] individuals) (Table 1). In adjusted models, odds of ever experiencing inequity in either written or verbal assessment were significantly higher among residents with a disability (odds ratio [OR], 7.80; 95% CI, 5.88-10.36) and those identifying as Asian (OR, 2.22; 95% CI, 1.27-3.87), URIM (OR, 6.77; 95% CI, 4.42-10.36), IMG (OR, 4.63; 95% CI, 2.28-9.40), or female (OR, 2.70; 95% CI, 1.95-3.73) than residents without a disability, White residents, or male residents (Table 2). Residents identifying as lesbian, gay, bisexual, queer, or other sexual orientation (OR, 0.61; 95% CI, 0.48-0.78) reported significantly lower odds of ever experiencing inequities than other identities. The intersectional interactions of URIM-female (OR, 0.44; 95% CI, 0.27-0.70) and Asian-female (OR, 0.50; 95% CI, 0.28-0.88) resulted in lower odds of ever experiencing inequities than odds for individual female, URIM, or Asian identities. We found similar results in residents reporting frequently experiencing inequity in assessment (Table 2).

**Table 1.** Demographic Characteristics of Pediatric Residents in 15 Programs Responding to a Multisite Survey

Characteristic	Residents, No. (%)		Pearson χ^2^, unadjusted *P* value^a^
	All (N = 479)	Reporting sometimes or usually experiencing inequity in assessment (n = 83)	
Race and ethnicity^b^			
Asian	108 (22.6)	20 (18.5)	.004
URIM^c^	82 (17.1)	24 (29.3)	
White	289 (60.3)	39 (13.5)	
Gender identity			
Female	345 (72.0)	73 (21.2)	<.001
Male	134 (28.0)	10 (7.5)	
Transgender, nonbinary, and other^d^	NA^e^	NA	
Sexual orientation			
Lesbian, gay, bisexual, queer, and other^f^	56 (11.7)	6 (10.7)	.16
Straight or heterosexual	423 (88.3)	77 (18.2)	
Disability identity			
With disability	39 (8.1)	13 (33.3)	.005
Without disability or did not report	440 (91.9)	70 (15.9)	
Medical degree			
IMG	25 (5.2)	8 (32.0)	.06
AMG	454 (94.8)	75 (16.5)	
SES			
Low^g^	110 (23.0)	28 (25.5)	.04
Middle^h^	264 (55.1)	40 (15.2)	
High^i^	105 (21.9)	15 (14.3)	

Abbreviations: AMG, American medical graduate; IMG, international medical graduate; NA, not applicable; SES, socioeconomic status; URIM, underrepresented in medicine.

^a^ Two-sided *P* < .05 indicates statistical significance.

^b^ Residents could self-identify with more than 1 race and ethnicity.

^c^ Includes African American or Black, American Indian or Alaskan Native, Hispanic, and Native Hawaiian or Other Pacific Islander.

^d^ Other gender identity includes genderqueer, gender nonconforming, and other (write-in).

^e^ Value too small to analyze.

^f^ Other sexual orientation includes pansexual and other (write-in).

^g^ Includes Pell grant recipient, free or reduced lunch program eligibility, and childhood family income less than $50 000 per year.

^h^ Includes childhood family income of $50 000 to $250 000 per year.

^i^ Includes childhood family income above $250 000 per year.

**Table 2.** Experiences and Frequency of Inequity in Assessment Reported by Pediatric Residents

Group	Odds of ever experiencing inequity in assessment^a^		Odds of experiencing inequity in verbal assessment		Odds of experiencing inequity in written assessment		Odds of frequently experiencing inequity in assessment^b^	
	OR (95% CI)	*P* value^c^	OR (95% CI)	*P* value^c^	OR (95% CI)	*P* value	OR (95% CI)	*P* value^c^
Race and ethnicity								
Asian	2.22 (1.27-3.87)	.005	3.76 (1.88-7.50)	<.001	1.00 (0.28-3.54)	.99	1.27 (0.98-1.63)	.07
URIM^d^	6.77 (4.42-10.36)	<.001	5.59 (3.08-10.13)	<.001	13.06 (6.07-28.10)	<.001	3.78 (3.07-4.67)	<.001
White	1 [Reference]	NA	1 [Reference]	NA	1 [Reference]	NA	1 [Reference]	NA
Gender identity								
Male	1 [Reference]	NA	1 [Reference]	NA	1 [Reference]	NA	1 [Reference]	NA
Female	2.70 (1.95-3.73)	<.001	2.59 (1.67-4.01)	<.001	3.24 (1.70-6.16)	<.001	2.85 (2.51-3.24)	<.001
Intersectional group: URIM- female	0.44 (0.27-0.70)	.001	0.51 (0.26-1.01)	.05	0.19 (0.08-0.46)	<.001	0.75 (0.60-0.94)	.01
Intersectional group: Asian- female	0.50 (0.28-0.88)	.02	0.39 (0.19-0.80)	.01	0.81 (0.22-2.97)	.75	1.42 (1.08-1.85)	.01
Disability identity								
Without disability	1 [Reference]	NA	1 [Reference]	NA	1 [Reference]	NA	1 [Reference]	NA
With disability	7.80 (5.88-10.36)	<.001	5.87 (3.92-8.78)	<.001	9.11 (5.57-14.88)	<.001	3.59 (3.22-4.00)	<.001
Sexual orientation								
Straight or heterosexual	1 [Reference]	NA	1 [Reference]	NA	1 [Reference]	NA	1 [Reference]	NA
Lesbian, gay, bisexual, queer, and other^e^	0.61 (0.48-0.78)	<.001	0.82 (0.59-1.15)	.25	0.45 (0.28-0.73)	.001	0.72 (0.64-0.82)	<.001
Medical degree								
AMG	1 [Reference]	NA	1 [Reference]	NA	1 [Reference]	NA	1 [Reference]	NA
IMG	4.63 (2.28-9.40)	<.001	1.73 (0.60-5.02)	.31	9.34 (3.54-21.60)	<.001	1.91 (1.62-2.26)	<.001
SES								
High^f^	1 [Reference]	NA	1 [Reference]	NA	1 [Reference]	NA	1 [Reference]	NA
Middle^g^	1.29 (1.05-1.59)	.02	1.10 (0.82-1.47)	.53	1.89 (0.68-5.28)	.23	0.90 (0.82-0.98)	.02
Low^h^	1.24 (0.97-1.59)	.08	1.08 (0.77-1.52)	.66	1.03 (0.65-1.63)	.91	1.42 (1.28-1.57)	<.001

Abbreviations: AMG, American medical graduate; IMG, international medical graduate; NA, not applicable; OR, odds ratio; SES, socioeconomic status; URIM, underrepresented in medicine.

^a^ Reporting experiencing inequity in either verbal or written assessment.

^b^ Reporting sometimes or usually experiencing inequity in assessment.

^c^ Two-sided *P* < .05 indicates statistical significance.

^d^ Includes African American or Black, American Indian or Alaskan Native, Hispanic, and Native Hawaiian or Other Pacific Islander.

^e^ Other sexual orientation includes pansexual and other (write-in).

^f^ Includes childhood family income above $250 000 per year.

^g^ Includes childhood family income between $50 000 and $250 000 per year.

^h^ Includes Pell grant recipient, free or reduced lunch program eligibility, and childhood family income below $50 000 per year.

## Discussion

Pediatric residents with a disability and those identifying as URIM reported 7- to 8-fold greater odds of experiencing inequities in assessment than residents without a disability or White residents. Residents identifying as IMG, Asian, or female also had greater odds of experiencing inequities, but to a lesser extent. Findings among URIM-female residents parallel findings from other specialties, indicating that race and ethnicity– and gender-based inequities are also a problem in pediatrics. The finding that URIM-female and Asian-female groups reported more experiences of inequity but not additive of levels reported by individual identities is similar to results in other specialties.^6^

A study limitation was our reliance on retrospective self-report. Future research is needed to confirm the findings and develop interventions to reduce inequities.

## Notes

Supplement 1. **eAppendix.** Survey Items

Supplement 2.
Data Sharing Statement
