# Supplementary material for: Inequity in Assessment Among Pediatric Residents
Source: JAMA Netw Open. 2025 Apr 17;8(4):e255594. doi: 10.1001/jamanetworkopen.2025.5594 (PMC12006863; doi:10.1001/jamanetworkopen.2025.5594)
Supplement: Supplement 1. — eAppendix. Survey Items [file jamanetwopen-e255594-s001.pdf]

Supplemental Online Content

Anderson HLK, West DC, Schwartz AJ, et al. Inequity in assessment among pediatric residents. *JAMA Netw Open*. 2025;8(4):e255594.
doi:10.1001/jamanetworkopen.2025.5594

eAppendix. Survey Items

This supplemental material has been provided by the authors to give readers additional information about their work.

© 2025 Anderson HLK et al. *JAMA Network Open*

eAppendix. Survey Items

Question	Response Options
| What year were you born?	| (Drop down) |
| In which country did you receive your medical degree?	| (Drop down) |
| Which of the following degrees have you obtained?	| • MD• DO• PhD• JD• MBA• MEd or MsEd• MPH• Other (please specify) |
| What is your current gender identity?[1]	| • Female• Male• Male-to-Female (MTF)/Transgender Female/Trans Woman• Female-to-Male (FTM)/Transgender Male/Trans Man• Nonbinary, Genderqueer, neither exclusively male nor female• Different identity (please state): _______• Prefer not to answer |
| What was your sex [assigned] at birth?[2]	| • Female• Male• Don't know/not sure• Prefer not to respond |
| Which of the following best represents how you think of yourself?[2]	| • Lesbian• Gay• Straight or heterosexual• Bisexual• Pansexual• Prefer to self-describe:• Don't know/not sure• Prefer to not respond |
| Which one or more of the following would you say is your race/ethnicity?[3]	| • American Indian or Alaska Native• Asian or Asian American• Black or African American• White• Hispanic, Latino, or Spanish Origin• Native Hawaiian or Other Pacific Islander• Different identity (please state): ________• Don't know/not sure• Prefer to not respond |
| [If Asian selected:] Which Asian origin best represents your identity?[3]	| • Indian• Vietnamese• Korean• Chinese• Filipino/a• Cambodian• Hmong• Japanese• Native Hawaiian and Pacific Islander• Don't know/not sure• Other (please state)• Prefer to not respond |
| [If Hispanic selected:] Which Hispanic, Latino, or Spanish origin best represents your identity?[2]	?| • Mexican• Puerto Rican• Cuban• Spanish• Another Hispanic, Latino/a, or Spanish origin (please specify:)• Don't know/not sure• Prefer to not respond |

© 2025 Anderson HLK et al. *JAMA Network Open*

| Are you a person with a disability?[4]	| • Yes• No• Don't know/not sure• Prefer to not respond |
| [If yes to disability:] Which of the following best describes your disability?[4]	| • ADHD• Chronic health condition(s)• Deaf or hard of hearing• Learning disability• Mobility disability• Psychological disability (including generalized anxiety and depression)• Visual disability• Other |
| Has your residency program provided accommodations for your disability?[4]	| • Yes• No |
| [If no to accommodations:] Which of the following best describes why?[4]	| • I have not requested because I feel I do not need accommodation(s)• My request for accommodations was denied• My request for accommodation is under review• I have not requested accommodation for other reasons (please specify:)• Prefer not to respond |
| During your childhood and adolescence (ages 0-18), what was the approximate annual income of your family?[3]	| • Less than $20,000• Less than $35,000• Less than $50,000• Less than $75,000• Less than $100,000• Less than $175,000• Less than $250,000• $250,000 or more• Don't know/not sure• Prefer to not respond |
| During your childhood and adolescence (ages 0-18), did you ever receive, or were you eligible for, free or reduced price lunch at school?[2]	| • Yes• No• Don't know/not sure• Prefer to not respond |
| Have you ever been a recipient of a Pell Grant?[3]	| • Yes• No• Don't know/not sure• Prefer to not respond |
| Are you among the first generation of your family to attend college or university?[3]	| • Yes• No• Don't know/not sure• Prefer to not respond |
| Are you among the first generation of your family to attend medical school?	| • Yes• No• Don't know/not sure• Prefer to not respond |
| Which of the following languages did you learn first?	| (Drop down) |
| Which of the following most accurately describes your background?	| • My parents/legal guardians and I were born in the U.S.• I was born in the U.S.; one parent/guardian was not• I was born in the U.S.; both of my parents/guardians were not• Foreign-born naturalized citizen• Permanent legal resident• Foreign born on student visa• Deferred Action for Childhood

© 2025 Anderson HLK et al. *JAMA Network Open*

	Arrivals (DACA) recipient• Refugee status• Prefer not to answer |
| Please indicate your religion or spiritual preference.[3]	| • Agnostic• Atheist• Christianity• Hinduism• Islam• Judaism• Other (please state):• Prefer to not respond |
| Have you ever experienced inequitable assessments because of your identity(ies) or background(s)? For each type, indicate if experienced due to:[3]	| Age• IMG status• Degree type• Gender Identity• Sex-assigned-at-birth• Sexual Orientation• Race/ethnicity• Disability• Socio-economic Status• First generation status• Citizenship status |
[if indicated] Please indicate the frequency you experienced these inequitable assessments:	| • Never• Rarely• Sometimes• Usually• Almost Always• Prefer to not respond |

eReferences

1. Association of Pediatric Program Directors (APPD). Guidance on Demographic Variables, 2021.
2. Centers for Disease Control and Prevention (CDC). Behavioral Risk Factor Surveillance System Survey Questionnaire. Atlanta, Georgia: U.S. Department of Health and Human Services, Centers for Disease Control and Prevention, 2020
3. Yemane, L., Kas-Osoka, O., Burns, A., Blankenburg, R., Prakash, L. K., Poitevien, P., Schwartz, A., Lucas, C. T., & Marbin, J. (2023). Upholding Our PROMISE: Underrepresented in Medicine Pediatric Residents' Perspectives on Interventions to Promote Belonging. *Academic medicine : journal of the Association of American Medical Colleges*, *98*(12), 1434–1442. https://doi.org/10.1097/ACM.0000000000005443
4. Association of American Medical Colleges (AAMC). 2023 Medical School Year Two Questionnaire, 2023.

Consent page shown to respondents:

Crosswalk Demographic Survey

We are conducting a study to determine the equity of reported EPA entrustment decisions and ACGME milestone assignments for residents. This short survey will collect demographic information about you.

As a research team, we are committed to advancing and ensuring equitable assessment for all learners, which is why we are asking you to self-report information that is relevant and important to you regardless of your identities or background. The survey is anonymous. We will keep the data we collect confidential, and we will not share the data with anyone outside the research team.

Some of the following questions may ask for sensitive details about you or your family; your responses will never be shared with your program or institution. If you are uncomfortable responding to any question, you can select "Prefer to not respond."

If you have questions about how we will use this data or how the study is being conducted, please contact Daniel Schumacher at daniel.schumacher@cchmc.org. If you have questions or concerns about your rights as a research participant, you can call the Cincinnati Children's Hospital Institutional Review Board at 513-636-8039.

There are 23 questions in this survey.

© 2025 Anderson HLK et al. *JAMA Network Open*
