# Supplementary material for: Inequity in Assessment Among Pediatric Residents
Source: JAMA Netw Open. 2025 Apr 17;8(4):e255594. doi: 10.1001/jamanetworkopen.2025.5594 (PMC12006863; doi:10.1001/jamanetworkopen.2025.5594)
Supplement: Supplement 2. — Data Sharing Statement [file jamanetwopen-e255594-s002.pdf]

Data Sharing Statement

Anderson. Inequity in Assessment Among Pediatric Residents. *JAMA Netw Open*. Published April 17, 2025. doi:10.1001/jamanetworkopen.2025.5594

Data
Data available: No
